# Implication of Exclusive Breastfeeding in Early Childhood Dental Disorders: Large Cohort Evidence, US National Survey of Children Health

**DOI:** 10.3390/children11101201

**Published:** 2024-09-30

**Authors:** Laurens Holmes, Janille Williams, Neyha Thompson, Valescia John, Kerti Depeika, Benjamin Ogundele, Michael Enwere

**Affiliations:** 1 Global Health Equity Foundation, Bear, DE 19701, USA; janillewilliams06@gmail.com (J.W.); neyah512@gmail.com (N.T.); vxjohn03@gmail.com (V.J.); keertikiyansh@gmail.com (K.D.); benjaminogundele@gmail.com (B.O.); mikky89@gmail.com (M.E.); 2 Lawhols International Scientific Research Consulting, Bear, DE 19701, USA; 3 Public Health & Allied Health Sciences Department, Delaware State University, Dover, DE 19901, USA; 4 School of Public Health, SUNY Downstate Health Sciences University, Brookyn, NY 11203, USA

**Keywords:** breastfeeding, dental disorders, early childhood caries (ECC)

## Abstract

**Purpose/Objective:** Frequent consumption of fermented carbohydrates and bottle feeding contribute to the development of early childhood caries (ECC). There are no substantial findings on the effects of breastfeeding patterns on oral health conditions in children in the United States. We aimed to assess the nexus between exclusive breastfeeding during the first 6 months and oral health disorders, namely toothache, decayed teeth, or unfilled cavities in early childhood. **Materials and Methods: Design:** Cross-sectional epidemiologic design of nationally representative data collected via telephone surveys in the United States was utilized. **Setting:** National Survey of Children’s Health, 2012 **Participants:** Population-based random sample of parents/guardians of 24,655 children aged 6 months to 5 years. **Main Outcome and Measure:** The primary outcome was the presence of oral health disorders (OHD) in children, defined as the parent-reported occurrence of one or more of the following conditions in the past 12 months: toothache, decayed teeth, or an unfilled cavity. This was assessed through a single composite question in the survey, asking parents/guardians if their child had experienced any of these specific oral health issues within the previous year with a binary (yes/no) response option. **Results:** Among 24,655 children aged 6 months to 5 years, 2392 (9.7%) had experienced an oral health disorder (OHD) in the previous 12 months. In unadjusted analysis, children exclusively breastfed for 6 months were 28% less likely to present with OHD compared to never-breastfed children (OR = 0.72, 95% CI, 0.52–0.98). However, after adjusting for key factors, including maternal health and race/ethnicity, this association was no longer statistically significant (aOR = 1.11, 95% CI 0.79–1.57). Significant predictors of OHD included maternal health (aOR for fair/poor vs. excellent/very good maternal health = 1.79, 95% CI 1.08–2.69) and race/ethnicity, with a higher prevalence among non-Hispanic Black (12.9%) and multi-racial children (12.6%) compared to non-Hispanic White children (7.7%). **Conclusions and Relevance:** While exclusive breastfeeding for the first six months of life was not found to be a significant predictor of pediatric oral health outcomes after adjustment, maternal health and race/ethnicity were significant determinants of oral health disorders. These findings underscore the importance of comprehensive healthcare approaches that consider maternal well-being and socio-demographic factors. Future research should explore interventions targeting these factors to improve pediatric oral health outcomes.

## 1. Introduction

Early childhood caries (ECC) encompasses a range of oral health conditions caused by bacteria that affect the structural architecture of primary teeth in children younger than 71 months. Oral health conditions classified as dental caries include tooth decay, cavities, and caries. In the United States, it is estimated that approximately 23% of children aged 2–5 years have dental caries in their primary teeth [1]. ECC is among the leading causes of chronic diseases among children, affecting more children than asthma, hay fever, or bronchitis [2,3]. Further, ECC is 5 times more common than asthma, one of the most recognized chronic health conditions among children [4].

The etiology and severity of ECC are primarily caused by microbial diseases but are also impacted by feeding and oral cleaning practices. *Streptococcus sobrinus* and *S. mutans* are the primary carcinogenic microorganisms responsible for tooth decay. Studies have shown that these bacterial pathogens occupy nearly 30% of the plaque flora in children with ECC [5,6]. Frequent consumption of fermented carbohydrates and bottle feeding also contribute to ECC development [7].

The World Health Organization recommends exclusive breastfeeding for the first six months of life and breastfeeding; afterward, they should be introduced to nutritious complementary foods while continuing breastfeeding until at least two years of age or longer [8]. Studies have demonstrated the nutritional, social, and economic benefits of breastfeeding in reducing the risk of bacterial, cardiovascular, and respiratory conditions. As new data emerge, our understanding of the importance and function of bacteria beyond their pathogenic properties expands. Despite these findings, breastfeeding has been noted as a potential risk factor for oral health diseases, specifically ECC [9,10]. Furthermore, the American Academy of Pediatric Dentistry cites nocturnal bottles and breastfeeding as potential risk factors for ECC. However, more recent studies have presented conflicting results regarding the role of breastfeeding and its duration on ECC risk [11,12].

To our knowledge, no study has assessed the association between breastfeeding and pediatric oral health problems using nationally representative data from the United States. Additionally, it is unknown whether this association varies by race. We aimed to characterize racial/ethnic heterogeneity in breastfeeding practices, as it relates to oral health disorders in early childhood, using national data. Specifically, we sought to assess the effects of exclusive breastfeeding during the first six months of life on the development of oral health disorders, mainly toothache, decayed teeth, or unfilled cavities, stratified race/ethnicity, namely Hispanics, non-Hispanic whites, and non-Hispanic blacks.

## 2. Materials and Methods

### 2.1. Data Source

We conducted a retrospective analysis of data collected from the National Survey of Children’s Health (NSCH) between 2011 and 2012. A secondary data analysis was performed using the NSCH data to assess the association between infant feeding practices and childhood oral health disorders. A nationally represented sample across the U.S. was created using randomly sampled telephone calls to capture the health and social experiences of non-institutionalized children aged 0–17 years. An adult family member with the most knowledge of a randomly selected child living in a household was queried. The surveys included questions about children’s health and well-being, in addition to information regarding family- and neighborhood-level demographic characteristics. Further details regarding the methodology and sampling technique have been described elsewhere [13].

This study utilized data from the National Survey of Children’s Health (NSCH) conducted in 2011–2012. This survey was designed to provide national and state-level estimates of various aspects of children’s health and well-being. The original NSCH sample included 95,677 child-guardian pairs aged 0–17 years. However, for this specific analysis focused on breastfeeding and early childhood oral health, the researchers restricted their sample to 24,655 children aged 6 months to 5 years. This age range was selected because it aligns with the period when exclusive breastfeeding typically occurs and when early childhood caries (ECC) can develop. The patient selection process for the NSCH involves a complex, stratified random sampling design to ensure a nationally representative sample. Households across the United States were randomly contacted via telephone surveys. In each selected household with children, one child was randomly chosen as the subject of the interview. An adult family member (usually a parent or guardian) most knowledgeable about the selected child’s health and health care was interviewed.

It is important to note that this study was a cross-sectional analysis of the NSCH data, not a longitudinal study with follow-up periods. The data were collected at a single time point for each participant, providing a snapshot of the child’s health status, breastfeeding history, and oral health outcomes. There was no active monitoring or follow-up of the patients over time. Instead, the study relied on retrospective reporting by parents/guardians about their child’s breastfeeding history and current oral health status.

The lack of longitudinal follow-up is a limitation of this study design, as it does not allow for the direct observation of how breastfeeding practices influence oral health outcomes over time. However, this approach does enable researchers to analyze a large, nationally representative sample, providing valuable insights into population-level associations between breastfeeding practices and oral health outcomes. Future research using prospective cohort designs could provide more robust evidence of the causal relationships between early feeding practices and subsequent oral health outcomes in children.

Given the design of our study and the data source (secondary data analysis of publicly accessible data), IRB approval was not necessary and informed consent was not required.

### 2.2. Study Population

The original study collected information from 95,677 child-guardian pairs aged 0–17 years. Given the focus on exclusive breastfeeding to six months, our analysis is restricted to 24,655 children aged 6 months—5 years old, representing over 19.7 million children. (Parents/guardians of children older than 5 years of age were not asked questions about early childhood including breastfeeding exposure.)

### 2.3. Variable Selection/Ascertainment

#### 2.3.1. Dependent Variable (Outcome)

The primary outcome variable in this study was oral health problems characterized by the presence of toothache, decayed teeth, or unfilled cavities. For children aged 6 months–5 years with natural teeth, respondents were asked about their history of toothache, decayed teeth, or unfilled cavities in the past 12 months.

Did [S.C.] have a toothache, decayed teeth, or unfilled cavities? Survey responses included “Yes”, “No”, and “Don’t Know”.

#### 2.3.2. Independent Variable

The primary independent variable was breastfeeding or being fed breast milk. This variable was constructed from responses to four questions regarding breastfeeding patterns and duration, resulting in three categories: never breastfed, breastfed (or fed breast milk) exclusively for six months, and non-exclusively breastfed (or fed breastmilk).

Given that the purpose of this study was to assess the effect of breastfeeding on oral health, participants who did not provide information about exposure to oral health problems or breastfeeding patterns were excluded from the sample.

### 2.4. Variables Selection

#### 2.4.1. Confounding Variables

Potential confounding variables included the following:

1. Demographic factors:
  - Race/ethnicity (non-Hispanic white, non-Hispanic black, Hispanic, non-Hispanic multiracial/other)
  - Sex
2. Socioeconomic factors:
  - Maternal education (less than high school, high school graduate, more than high school)
  - Poverty index (DHHS Poverty Level)
  - Health insurance type (private, public, currently uninsured)
3. Health-related factors:
  - Maternal health status (excellent/very good, good, fair/poor)
  - Receipt of dental care
  - Unmet dental needs

The study variables were race/ethnicity, maternal education (less than high school, high school graduate, more than high school), poverty index, health insurance type (private, public, currently uninsured), and maternal health status (excellent/very good, good, fair/poor). Given that specific information on dental insurance was not collected in the 2011 version of the NSCH survey, access to health insurance was used as a proxy for dental insurance coverage.

#### 2.4.2. Additional Variables

Additional variables related to dental health care utilization were also included:

- Dental service utilization in the past 12 months
- Prevalence of unmet dental needs

Additional covariates used in the analysis included receipt of dental care and unmet dental needs. To estimate dental service utilization, parents/guardians were asked, “During the past 12 months, did the child see a dentist for any kind of dental care, including check-ups, dental cleanings, X-rays, or filling cavities?”. To assess the prevalence of unmet dental needs participants were asked, “During the past 12 months, was there any time when the child needed [dental] care but it was delayed or not received?”. Participants who responded “No” to either question were classified as not utilizing dental care services or having an unmet dental need.

Suspected confounding variables were selected a priori based on known predictors of poor oral health among children. To account for the diversity of experiences in racial/ethnic groups, we computed a combined four-category race/ethnicity variable using the information collected on racial and ethnic identification in the NSCH survey. Those who were identified as Hispanic/Latino were categorized as such in the computed variables. All other participants were categorized as non-Hispanic and one of the three racial categories (non-Hispanic white, non-Hispanic black, and non-Hispanic multiracial/other), as reported by parents/guardians.

### 2.5. Statistical Analyses

Categorical and nominal variables were summarized using frequencies and percentages. To assess the association between exclusive breastfeeding and oral health outcomes, logistic regression models were used to determine the effects of demographic characteristics on oral health problems. Unconditional multivariable logistic regression models were used to assess the association between breastfeeding patterns and oral health disorders, adjusting for the effects of race/ethnicity, maternal education, poverty index, insurance coverage, unmet dental needs, dental care receipt, and maternal health status. Finally, forward loading and backward elimination were employed to characterize the final model. To determine the precision measure on the association between breastfeeding and oral health problems, the confidence interval (CI) was set at 95%, while the statistical significance level was set at 0.05 (5%) as type I error tolerance for univariable analysis. Additionally, the precision measure for the multivariable assessment was set at 99% CI, whereas the type-I error tolerance was set at 1% (0.01). All tests were two-tailed, and STATA Statistical Software, Version 17.0 (STATA Corp, College Station, TX, USA) was used in all analyses.

## 3. Results

Table 1 provides a detailed breakdown of the study characteristics stratified by race/ethnicity, highlighting differences in breastfeeding practices, maternal education levels, and oral healthcare access among different racial groups.

Among the children included in our final sample, 19,145 (77.6%) were breastfed (or fed breast milk) and 4117 (16.7%) were breastfed exclusively for the first 6 months (Table 1). Approximately 9.7% (n = 2391) of the children had experienced a toothache, decayed teeth, or unfilled cavity in the past 12 months.

In the unadjusted analysis (Table 2), breastfeeding was found to be a protective factor against poor oral health. Children breastfed exclusively for the first 6 months were 28% less likely to have an oral health problem in the past 12 months when compared to children who were never breastfed (or fed breast milk), OR = 0.72, 95% CI 0.52–0.98.

However, after adjusting for health insurance, maternal education and overall health, unmet dental needs, and dental care receipt (Table 3), the protective nature of breastfeeding on oral health problems did not persist. In the multivariable model, exclusive breastfeeding at six months was not associated with toothache, decayed teeth, or unfilled cavities (adjusted odds ratio [aOR] = 1.11, 99% CI 0.71–1.75). Similarly, non-exclusive breastfeeding showed no significant association with oral health outcomes (aOR = 1.04, 99% CI 0.75–1.46).

The adjusted models (Table 3) identified several other factors as significant predictors of oral health problems:

- Race/ethnicity: Non-Hispanic black (aOR = 1.60, 99% CI 1.03–2.50) and non-Hispanic multiracial/other children (aOR = 1.79, 99% CI 1.18–2.72) had an increased risk for oral health problems compared to non-Hispanic white children.
- Maternal health: Children of mothers with fair/poor health had a 70% increased risk for poor oral health compared to children of mothers with excellent/very good health (aOR = 1.70, 99% CI 1.08–2.69).
- Unmet dental needs: Children with unmet dental needs were 8 times more likely to have an oral health problem compared to children who did not experience delays in dental care (aOR = 8.20, 99% CI 3.56–18.85).
- Dental care: Children who had not received any dental care in the past 12 months had an 86% reduced risk of oral health problems (aOR = 0.14, 99% CI 0.09–0.20).

These findings suggest that while breastfeeding practices were not significantly associated with oral health outcomes in the adjusted models, other factors, such as race/ethnicity, maternal health, and access to dental care, play crucial roles in determining oral health outcomes in early childhood (Table 3).

## 4. Discussion

This study aimed to assess the association between exclusive breastfeeding (during the first 6 months of life) and later adverse oral health outcomes in early childhood. Given the magnitude of the data, the U.S. The 2012 National Survey of Children’s Health was used to perform a retrospective analysis. Our findings suggest that breastfeeding (or the consumption of breast milk) is not associated with oral health problems in young children. Additionally, the data implicate maternal health as a significant predictor of oral health problems in early childhood and suggest that an estimated 8.9 million children in the United States potentially have undiagnosed and untreated poor oral health, posing an undue burden, especially on those who are most vulnerable.

Poor oral health is associated with increased school absences, poor school performance, and reduced quality of life [4,14]. Jackson et al., in their study of the impact of oral health on school performance among school children in North Carolina, found that relative to children with excellent and very good oral health status, children with good, fair, or poor oral health status were almost 3 times more likely to miss school due to dental pain [1]. Additionally, Jackson et al. found an independent association between oral health status and school performance, where children with good, fair, or poor oral health were more likely to underperform in school when compared to children with excellent or very good health status [1].

Our findings showed that the prevalence of breastfeeding was greatest in non-Hispanic whites and lowest in non-Hispanic blacks. This finding is consistent with the data presented in previous studies. Even after adjusting for known risk factors, the association between race/ethnicity and oral health problems remained for non-Hispanic Black and multiracial/other children. An analysis of the 2003 NSCH data found that regardless of dental insurance type or income, black children were less likely to use preventive dental care services [15]. Conversely, this may explain why the association between Hispanic ethnicity and oral health problems disappeared in the multivariable models. These findings suggest that the causal pathway for poor oral health is different for racial/ethnic groups, and perhaps health insurance best explains the observed association between Hispanic ethnicity and poor oral health in bivariate models.

We estimated the prevalence of oral health problems (toothache, decayed teeth, and unfilled cavities) to be approximately 9.6% of the pediatric population, affecting more than 2.1 million children in the United States. Further, we estimated that approximately 8.9 million children did not receive dental care services in the last year. The American Academy of Pediatric Dentistry recommends oral hygiene counseling and caries risk assessment every 6 months.

Maternal health was observed to be an independent indicator of poor oral health, with children raised by mothers with excellent or very good health having the lowest risk of developing poor oral health. This finding is consistent with previously published data on the influence of maternal health on pediatric oral health outcomes. Some studies have indicated that maternal depression may have an impact on maternal awareness of child health and subsequent healthcare utilization. This may be impacted by the mother’s prioritization of dental health needs in relation to seemingly more important tasks and responsibilities.

Children with public insurance were more likely to have oral health disorders than children with private insurance. Consistent with the literature, we observed a dose-response relationship between poverty level and oral health disorders, where children in the fourth quintile were least likely to have toothaches, decayed teeth, or caries as compared to children in the first quartile. It is likely that children from lower socioeconomic backgrounds are less likely to have health or dental insurance or experience difficulties in covering any out-of-pocket expenses related to dental care services.

This study had several limitations. All the data for NSCH were collected from parents/guardians at a single time point; therefore, we cannot assert temporality between some of the health-seeking behaviors and the primary outcome—oral health problems. For the primary research question, this is not of concern because for most children, breastfeeding exposure is time bound to the first year of life, occurring before the development of caries. In addition, beginning with the 2007 NSCH survey, data were no longer collected for dental insurance. In our study, we used health insurance coverage as a proxy for dental insurance, recognizing the potential overestimation of insurance coverage for the sample. Despite these concerns, some data show that health insurance is an appropriate proxy measure for dental insurance. Under Medicaid and the Children’s Health Insurance Program (CHIP), comprehensive dental care visits are free for children.

In summary, health insurance, maternal education and health, minority racial identification, and unmet dental needs predicted oral health disorders in the study sample. It is important to acknowledge the potential biases that may have influenced our findings. Information bias, particularly recall bias, was a significant concern in this study. Parents/guardians were asked to report on breastfeeding practices that occurred several years prior to the survey, which may have led to inaccurate recollections, especially regarding the duration and exclusivity of breastfeeding. Additionally, the self-reported nature of oral health problems might be subject to under- or over-reporting, depending on parents’ awareness and interpretation of their children’s oral health status. This reliance on self-reported data could lead to misclassifications or measurement errors, particularly when asked about dietary habits. The use of telephone surveys for data collection introduces the possibility of sampling bias. Households without landline telephones or those that primarily use cell phones may have been underrepresented, potentially affecting the generalizability of our findings. This sampling approach might have led to an underrepresentation of certain demographic groups, such as younger parents or those from lower socioeconomic backgrounds, who are more likely to rely solely on cell phones. Furthermore, respondents willing to participate in telephone surveys may differ systematically from those who do not, potentially limiting the generalizability of our findings. The cross-sectional nature of the data also limited our ability to establish causal relationships between breastfeeding practices and oral health outcomes. Despite these limitations, the large sample size and the use of nationally representative data strengthen the validity of our findings. Future studies employing prospective designs and more objective measures of oral health status could help address these potential biases and further elucidate the relationship between breastfeeding and early childhood oral health. These biases should be carefully considered when interpreting the study results. This is the largest study to determine the association between breastfeeding and oral health problems in children using nationally representative data from the United States. Our findings suggest that approximately 9 million children in the United States may not be receiving the recommended dental care in the form of dental cleaning, oral hygiene counseling, and caries risk assessments. It’s also worth noting that anatomical factors, such as ankyloglossia (tongue-tie) due to a short lingual frenulum, can interfere with breastfeeding [16]. This condition may lead some mothers to discontinue breastfeeding early if not properly diagnosed and treated. Even with Medicaid and CHIP expansion and increased dental care coverage, millions of children do not receive recommended oral health assessments and care. An analysis of 2012 data from the most reliable data source for national dental-care utilization found that 39.5% of children with public insurance received dental care [17].

Our study provides critical insights into the relationship between oral health and children’s overall well-being, particularly in the context of school performance, absenteeism, and early childhood health. We found a strong correlation between poor oral health, increased school absences, and lower academic achievement. This emphasizes the importance of early and effective oral health interventions, particularly in underserved communities, to address their negative impacts on educational outcomes and quality of life. Additionally, this large-scale, nationally representative study examined the relationship between breastfeeding practices and early childhood oral health in the United States. Contrary to some concerns, our findings suggest that exclusive breastfeeding for the first six months of life is not associated with an increased risk of oral health problems. However, significant predictors of poor oral health outcomes were identified, including racial/ethnic disparities, maternal health status, and access to dental care. Notably, children with unmet dental needs face a substantially higher risk of oral health problems, underscoring the importance of early and regular dental care. These results highlight the need for targeted interventions to address disparities in oral health care access, particularly among minority and socioeconomically disadvantaged populations, while also emphasizing the critical role of maternal health in pediatric oral health outcomes. Comprehensive public health policies that integrate oral health programs within schools and combine maternal and child health care approaches could have significant benefits for both academic success and long-term health outcomes.

## Figures and Tables

**Table 1:** Characteristics of Study Participants by Race/Ethnicity.

Variable	Race			
	White, Non-Hispanic	Black, Non-Hispanic	Hispanic	Multi-Racial/Other, Non-Hispanic
**Sex**	n (%)	n (%)	n (%)	n (%)
Male	7614 (50.8)	1139 (50.9)	1957 (51.7)	1523 (50.0)
Female	7385 (49.2)	1098 (49.1)	1829 (48.3)	1525 (50.0)
**Breastfeeding**				
Never Breastfed	3080 (20.5)	861 (38.4)	748 (19.8)	690 (22.6)
Exclusive 6 months	2.674 (17.8)	240 (10.7)	587 (15.5)	518 (17.0)
Breastfed, Non-Exclusively	9254 (61.7)	1139 (50.9)	2453 (64.8)	1843 (60.4)
**Maternal Education**				
Less than High School	548 (3.8)	178 (8.8)	934 (26.2)	201 (7.0)
High School Graduate	2173 (15,2)	485 (23.9)	900 (25.2)	556 (19.4)
More than High School	11,566 (81.0)	1365 (67.3)	1738 (48.7)	2112 (73.6)
**Unmet Dental Needs**				
No	14,821 (98.8)	2198 (98.3)	3739 (98.8)	2990 (98.2)
Yes	179 (1.2)	38 (1.7)	44 (1.2)	56 (1.8)
**Mother’s Mental/Emotional Health**				
Excellent/Very Good	11,475 (80.2)	1446 (71.2)	2401 (66.7)	2140 (74.5)
Good	2209 (15.5)	422 (20.8)	917 (25.5)	557 (19.4)
Fair/Poor	616 (4.3)	164 (8.1)	281 (7.8)	177 (6.2)
**Insurance Type Coverage**				
Private	10,694 (71.8)	781 (35.5)	1332 (35.8)	1697 (56.9)
Public	3740 (25.1)	1342 (60.9)	2171 (58.3)	1155 (38.7)
Currently Uninsured	451 (3.0)	79 (3.6)	220 (5.9)	130 (4.4)
**DHHS Poverty Level**				
<99% FPL	1551 (11.1)	672 (32.9)	1291 (37.5)	626 (22.2)
100–199% FPL	2395 (17.1)	487 (23.8)	878 (25.5)	548 (19.4)
200–399% FPL	4682 (33.4)	497 (24.3)	703 (20.4)	714 (25.3)
≥400% FPL	5377 (38.4)	388 (19.0)	569 (16.5)	934 (33.1)
**Oral Health Disorders**				
No	13,860 (92.4)	1951 (87.1)	3284 (86.7)	2666 (87.4)
Yes	1148 (7.7)	289 (12.9)	504 (13.3)	385 (12.6)
**Oral Health Prevention**				
1 or more preventive care	8187 (54.6)	1362 (61.0)	2175 (57.6)	1611 (53.0)
No Preventive Visits	6797 (45.4)	871 (39.0)	1602 (42.4)	1429 (47.0)
**Oral Health Care**				
Received dental care	8250 (55.0)	1374 (61.4)	2197 (58.1)	1639 (53.8)
No dental care	6740 (45.0)	865 (38.7)	1584 (41.9)	1407 (46.2)

**Table 2:** Association Between Breastfeeding Practices and Oral Health Outcomes.

Variable	Oral Health Disorders (Toothache, Decayed Teeth, Unfilled Cavity)			
	Yes	No	X^2^ (df)	*p*
**Race/Ethnicity**			184.8 (3)	<0.001
White, non-Hispanic	1148 (7.7)	13,860 (92.4)		
Black, non-Hispanic	289 (12.9)	1951 (87.1)		
Hispanic	504 (13.3)	3284 (86.7)		
Multi-racial/Other, non-Hispanic	385 (12.6)	2666 (87.4)		
**Sex**			7.0 (1)	0.008
Male	1275 (53.3)	11,233 (50.5)		
Female	1116 (46.7)	11,022 (49.5)		
**Breastfeeding**			53.0 (2)	<0.001
Never Breastfed	676 (28.3)	4844 (21.8)		
Exclusive 6 months	377 (15.8)	3740 (16.8)		
Breastfed, Non-Exclusively	1339 (56.0)	13,689 (61.5)		
**Maternal Education**			314.4 (2)	<0.001
Less than High School	388 (15.7)	1536 (7.4)		
High School Graduate	550 (25.6)	3587 (17.3)		
More than High School	1260 (58.7)	15,657 (75.4)		
**Unmet Dental Needs**			303.1 (1)	<0.001
No	2265 (94.8)	22,051 (99.1)		
Yes	124 (5.2)	202 (0.9)		
**Mother’s Mental/Emotional Health**			129.5 (2)	<0.001
Excellent/Very Good	1461 (66.9)	16,250 (77.5)		
Good	533 (24.4)	3655 (17.4)		
Fair/Poor	190 (8.7)	1069 (5.1)		
**Insurance Type Coverage**			345.5 (2)	<0.001
Private	1017 (43.2)	13,780 (62.7)		
Public	1234 (52.4)	7413 (33.7)		
Currently Uninsured	104 (4.4)	801 (3.6)		
**DHHS Poverty Level**			310.1 (3)	<0.001
<99% FPL	648 (29.6)	3520 (17.4)		
100–199% FPL	536 (24.5)	3809 (18.8)		
200–399% FPL	551 (25.1)	6080 (30.0)		
≥400% FPL	457 (20.9)	6865 (33.9)		
**Oral Health Prevention**			1.200 (1)	<0.001
1 or more preventive care	2114 (88.5)	11,557 (52.0)		
No Preventive Visits	274 (11.5)	10,668 (48.0)		
**Oral Health Care**			1.200 (1)	<0.001
Received dental care	2128 (89.0)	11,672 (52.5)		
No dental care	263 (11.0)	10,571 (47.5)		

**Table 3:** Adjusted Odds Ratios for Oral Health Outcomes by Breastfeeding Duration.

Variable	Model I	Model II	Model III
	Odds Ratio (95% CI)	Adjusted Odds Ratio (99% CI)	Adjusted Odds Ratio (99% CI)
**Race/Ethnicity**			
White, non-Hispanic	1.00	1.00	1.00
Black, non-Hispanic	2.01 (1.54–2.64) ***	1.60 (1.03–2.50) **	1.54 (0.99–2.38) *
Hispanic	2.21 (1.73–2.82) ***	1.10 (0.75–1.60)	1.08 (0.73–1.60)
Multi-racial/Other, non-Hispanic	1.70 (1.28–2.26) ***	1.79 (1.18–2.72) ***	1.82 (1.18–2.79) ***
**Sex**			
Male	1.00	1.00	1.00
Female	0.95 (0.78–1.16)	-	-
**Breastfeeding**			
Never Breastfed	1.00	1.00	1.00
Exclusive 6 months	0.72 (0.52–0.98) *	1.11 (0.71–1.75)	1.10 (0.70–1.74)
Breastfed, Non-Exclusively	0.73 (0.58–0.92) **	1.04 (0.75–1.46)	1.03 (0.73–1.46)
**Maternal Education**			
Less than High School	1.00	1.00	1.00
High School Graduate	0.64 (0.47–0.87) **	0.63 (0.41–0.97) *	0.59 (0.38–0.94) **
More than High School	0.35 (0.27–0.46) ***	0.39 (0.26–0.61) ***	0.40 (0.25–0.65) ***
**Unmet Dental Needs**			
No	1.00	1.00	
Yes	7.38 (4.58–11.9) ***	8.20 (3.56–18.85) ***	8.65 (3.62–20.7) ***
**Mother’s Health**			
Excellent/Very Good	1.00	1.00	1.00
Good	1.78 (1.41–2.24) ***	1.35 (0.96–1.92) *	1.35 (0.95–1.92) *
Fair/Poor	2.36 (1.75–3.17) ***	1.70 (1.08–2.69) **	1.51 (0.96–2.37) *
**Insurance Type Coverage**			
Private	1.00	1.00	1.00
Public	2.27 (1.86–2.77) ***	1.29 (0.92–1.82)	-
Currently Uninsured	2.28 (1.34–3.88) **	1.82 (0.83–3.97) *	-
**DHHS Poverty Level**			
<99% FPL	1.00	1.00	1.00
100–199% FPL	0.78 (0.60–1.03)	-	1.11 (0.74–1.67)
200–399% FPL	0.55 (0.41–0.73) ***	-	0.84 (0.52–1.35)
≥400% FPL	0.32 (0.25–0.42) ***	-	0.57 (0.36–0.91) **
**Oral Health Prevention**			
1 or more preventive care	1.00	1.00	1.00
No preventive visits	0.16 (0.12–0.21) ***	-	-
**Oral Health Care**			
Received dental care	1.00	1.00	1.00
No dental care	0.16 (0.12–0.21) ***	0.14 (0.09–0.20) ***	0.14 (0.09–0.21) ***

## Data Availability

This study applied the secondary data from the National Survey of Children’s Health with Data Used Approval to Professor Laurens Holmes, Jr.
